# Multitemporal single‐cell profiling decoding crosstalk between γδ17 T cells and neutrophils in radiation pneumonitis

**DOI:** 10.1002/ctm2.1700

**Published:** 2024-05-17

**Authors:** Wenting Ren, Xiaoxiang Zhou, Ziming Jiang, Shiqi Li, Haoxuan Zhang, Jianrong Dai, Yexiong Li, Nan Bi, Yibo Gao, Jie He

**Affiliations:** ^1^ Department of Thoracic Surgery, National Cancer Center/National Clinical Research Center for Cancer/Cancer Hospital Chinese Academy of Medical Sciences and Peking Union Medical College Beijing China; ^2^ Department of Radiation Oncology, National Cancer Center/National Clinical Research Center for Cancer/Cancer Hospital Chinese Academy of Medical Sciences and Peking Union Medical College Beijing China; ^3^ Central Laboratory and Shenzhen Key Laboratory of Epigenetics and Precision Medicine for Cancers, National Cancer Center/National Clinical Research Center for Cancer/Cancer Hospital and Shenzhen Hospital Chinese Academy of Medical Sciences and Peking Union Medical College Shenzhen China; ^4^ Laboratory of Translational Medicine, National Cancer Center/National Clinical Research Center for Cancer/Cancer Hospital Chinese Academy of Medical Sciences and Peking Union Medical College Beijing China; ^5^ State Key Laboratory of Molecular Oncology, National Cancer Center/National Clinical Research Center for Cancer/Cancer Hospital Chinese Academy of Medical Sciences and Peking Union Medical College Beijing China

Dear Editor,

Radiation pneumonitis (RP), a form of radiation therapy‐induced normal tissue toxicity, significantly hinders cancer treatment outcomes.^1^ The involvement of immune cells in RP is not completely understood. Using single‐cell RNA sequencing (scRNA‐seq) combined with bulk RNA sequencing (bulk RNA‐seq) and proteomics, we identified the accumulation of neutrophils and T cells and investigated their function in RP progression. These findings reveal intricate and evolving immunologic alterations in RP, offering insights for potential therapeutic strategies.

In the RP murine model, Mice were exposed to 20 Gy thoracic irradiation, with Day 10 representing the early and Day 100 the late phase of RP (Figure 1A). Classic RP symptoms were observed,^2^ including weight loss (Figure S1B), hair colour change (Figure S1C), computed tomography scan anomalies (Figure 1B), defective pneumocytes, widening of the alveolar septum, immunocyte infiltration in hematoxylin and eosin (H&E)‐stained slices (Figure 1C), and reduced lung function (Figure 1D). We pooled three mice per group and collected a total of 28,287 cells on days 0, 10, and 100 (Figure 1A). Dynamic cellular changes indicate an increase in the proportions of neutrophils (*Csf3r*) and T/B lymphocytes (*Cd3d*/*Cd79a*) (Figure 1E–G and Figure S1D,E), as verified by H&E staining (Figure 1C). Bulk RNA‐seq and proteomics confirmed elevated *S100a8*, *Ngp*, and *Retnlg* expression in neutrophils (Figure S1E–I).

**FIGURE 1:**
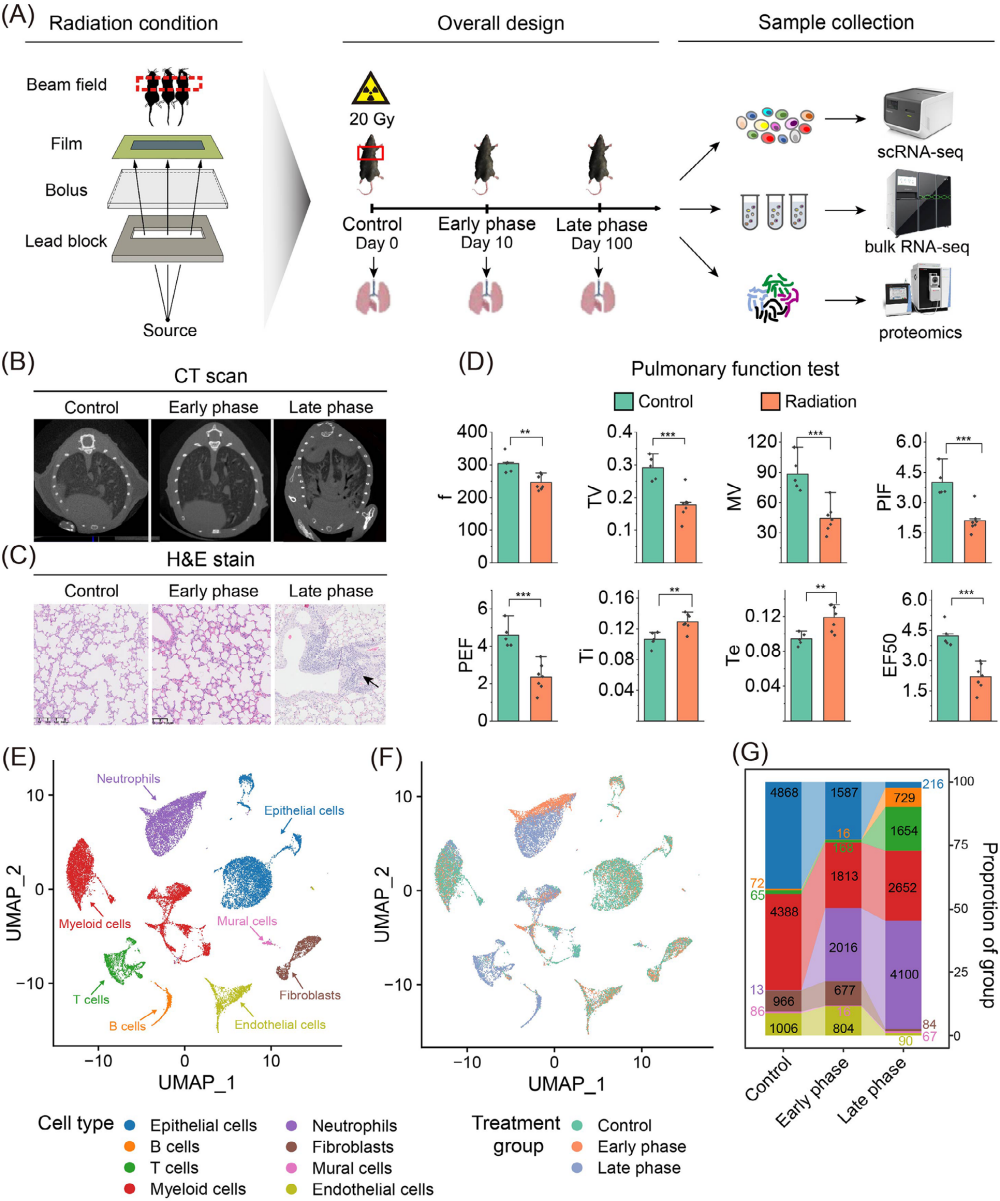
Experimental scheme and identification of cell types in radiation pneumonitis (RP). (A) Irradiation scheme. The 9 MeV electron ray passes through the applicator, lead block, bolus and film, and finally arrives at the thorax of mice. Mice were irradiated with a single dose of 20 Gy at day 0 and collected lung tissue at day 10 and day 100 after radiation therapy (RT) for single‐cell RNA sequencing (scRNA‐seq), bulk RNA‐seq, and proteomics. For each group, three mice were pooled to generate the scRNA‐seq data. (B) Computed tomography (CT) images of the thorax in control, early‐phase and late‐phase groups. (C) H&E staining of lung tissue in control, early‐phase and late‐phase groups. (D) Lung function tests in the early phase were measured using the Buxco non‐invasive airway mechanics plethysmograph. Mice were monitored and multiple ventilatory parameters were determined by the FinePointe software over a period of 10 min. Unpaired t‐test, **, *p*<.01, ***, *p* < .001. (E) Uniform Manifold Approximation and Projection (UMAP) plot of 28,153 cells for scRNA‐seq, colored by cell type annotations. (F) UMAP plot colored by time. (G) Histogram of cell type fractions of control, early‐phase and late‐phase group, colored by cell type. Control, untreated group; Early phase, 10 days after radiation; Late phase, 100 days after radiation.

Post‐radiation, neutrophil infiltration accumulated in pneumonitis and was confirmed by flow cytometry (Figure 2A and Figure S2A,B). We categorized neutrophils into five subsets [pre‐neutrophils (preNeu), immature neutrophils (immNeu), mature neutrophils (mNeu), Isg15^+^ neutrophils (Neu_Isg15), Gm2a^+^ neutrophils (Neu_Gm2a)] (Figure 2B), with preNeu and immNeu prevalent in the early phase, and mNeu, Neu_Isg15, and Neu_Gm2a in the late phase (Figure 2C). Gene expression analysis revealed primary granule genes (*Mgp* and *Camp*) highly expressed in preNeu and immNeu, while immune defence genes (*Ccl6*) and maturation genes (*Cxcr2* and *Cxcr4*) were prominent in mNeu, Neu_Isg15 and Neu_Gm2a (Figure 2D). Additionally, interferon‐stimulated genes (*Ifit1* and *Isg15*) were notably expressed in Neu_Isg15.^3^

**FIGURE 2:**
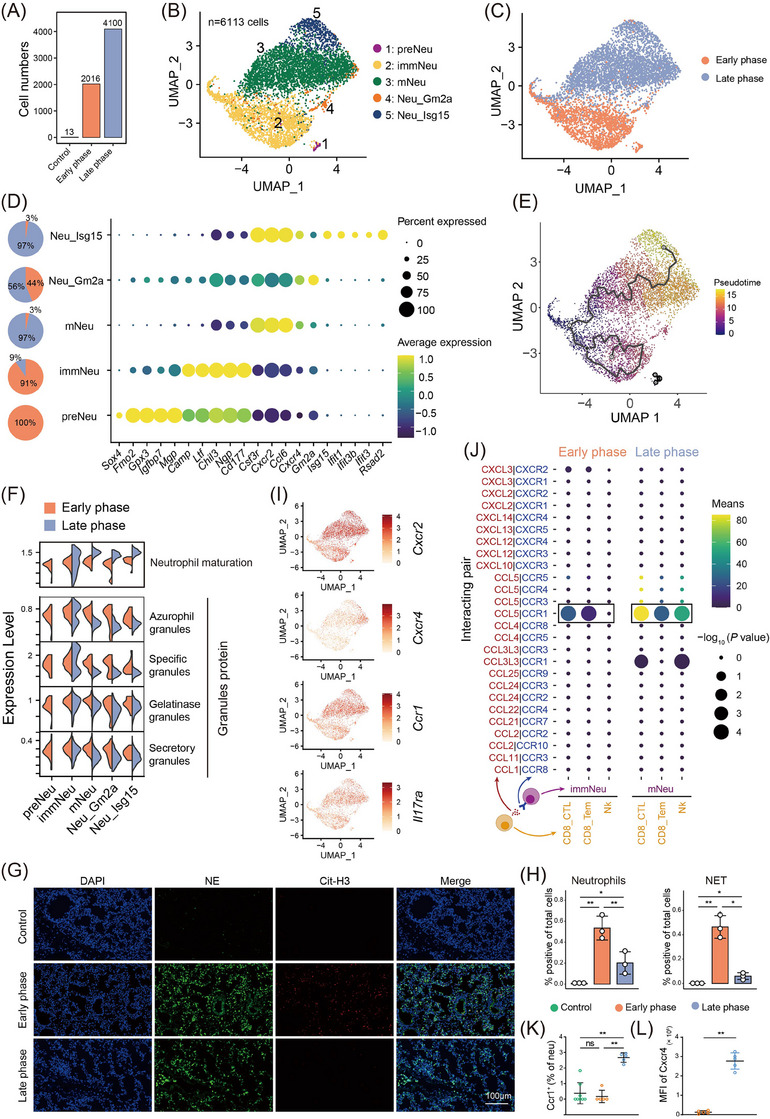
Distinct characteristics and trajectories of neutrophils in the two phases of radiation pneumonitis (RP). (A) Cell counting of neutrophils in control, early‐phase and late‐phase groups. (B) Louvain clustering of 6113 neutrophils into five subpopulations. (C) Uniform Manifold Approximation and Projection (UMAP) plot of neutrophils, coloured by different time points. (D) Markers of neutrophil subsets with a pie chart of neutrophil distribution. (E) Trajectory upon UMAP plot of neutrophils by monocle3. (F) Violin plots showing selected pathway scores for the early and late phase group. (G) Representative immunofluorescent images of neutrophil elastase (NE, green), citrullinated histone‐H3 (Cit‐H3, red) and DAPI (blue) stained lungs at early and late phase (*n* = 3 mice per group, each timepoint). Scale bar, 100 µm. (H) neutrophils (left panel) and NET signal (right panel) quantification (*n* = 3 mice, each with at least four technical replicates). (I) UMAP plots showing expression of selected receptor genes in neutrophil subsets. (J) Dot plot of ligand–receptor pair between neutrophils (yellow) and T cells (purple) in the early and late phase. (K, L) Quantification of Ccr1^+^ cells of neutrophils (K) and MFI of Cxcr4 (L) in the lungs of mice during the late phase RP, early phase RP, and in control mice by flow cytometry (*n* = 7 control mice, *n* = 5 irradiated mice at early and late phase). A two‐sided Wilcoxon test is adopted for comparing each group. ns, *p* ≥ 0.05, *p < .05, **p < .01 and ***p < .001. neu, neutrophil; MFI, mean fluorescence intensity; NET, neutrophil extracellular trap.

To investigate neutrophil roles in RP, we conducted transcription factor network analysis which revealed preNeu and immNeu enriched with stemness genes (*Gata2* and *Tbx4*), while mNeu and Neu_Isg15 expressed maturation‐related transcription factors (*Junb* and *Cebpd*) (Figure S2C). Differential trajectory analysis indicated distinct differentiation states between early and late‐phase neutrophils with no sequential development (Figure 2E). Examining the granule protein gene signature by Xie et al.,^3^ we found divergent granule protein expression in neutrophil phases (Figure 2F). Surprisingly, early‐phase neutrophils showed increased expression of all granule types. Considering the cytotoxic effects of granule contents in neutrophils,^4^, ^5^ we analyzed gene profiles related to cytotoxicity. The immNeu subset showed increased neutrophil extracellular trap (NET) and reactive oxygen species production, causing tissue damage (Figure S2D,E), confirmed by NET enrichment in the early phase (Figure 2G,H). Conversely, mNeu displayed enhanced chemotaxis and transcriptional activity, suggesting stronger cellular interactions (Figure S2D,E). This subset also showed elevated proinflammatory cytokines (*Cxcl3* and *Il1b*), implying persistent inflammation (Figure S2F). Further analysis revealed upregulated chemokine receptors (*Il17ra*, *Ccr1*, *Cxcr2* and *Cxcr4*) in late‐phase neutrophils (Figure 2I), with potential CD8^+^ T cell recruitment (Figure 2J,K and Figure S2G). Late‐phase RP also saw increased maturation markers (*Cxcr4* and *Cxcr2*), confirmed by flow cytometry (Figure 2L and Figure S2H).

To decipher the roles of T/NK cells in RP development, we classified T/NK cells into ten subsets (Figure 3A). The identity of each subset was validated by T/NK cell lineage and function marker genes (Figure S3A). All T/NK subsets were enriched in the late phase of RP (Figure 3B). Notably, a subset of γδT cells, marked by high Il17a expression and known as γδ17 T cells, was almost exclusive to the late phase (Figure 3C,D).^6^ Flow cytometry confirmed an increased proportion of interleukin (IL)17A^+^ γδT cells in late‐phase (Figure 3E,F and Figure S3B). Uniquely, we identified γδ17 T cells by the γδ chain variable region and revealed they belonged to a novel Vδ4^+^ subset and a common Vγ6^+^ subset (Figure 3F), previously noted only in a mouse skin infection model, underscoring the rarity of this γδ17 T cell type.^7^

**FIGURE 3:**
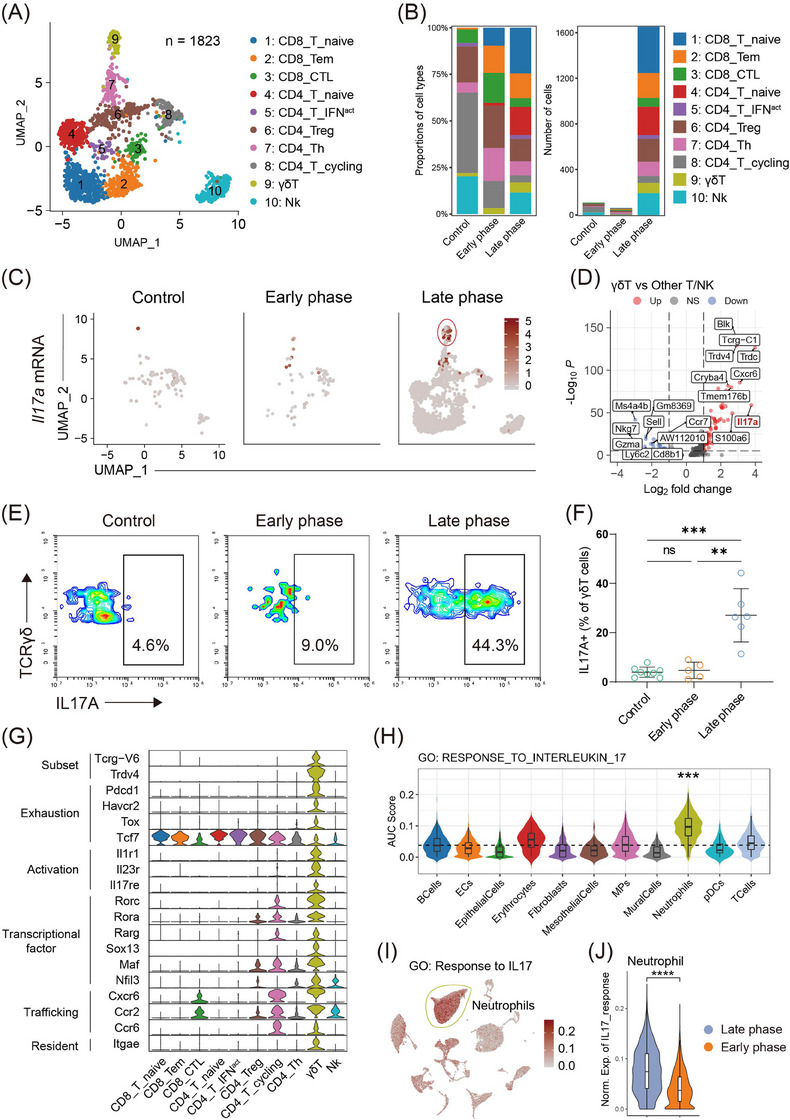
A rare Vγ6^+^Vδ4^+^ γδ17 T population may interact with neutrophils via the interleukin (IL)17A axis in the late phase of radiation pneumonitis (RP). (A) Identification of 10 sub‐clusters of T and natural killer (NK) cells. (B) The proportions (left) and frequencies (right) of T/NK sub‐clusters across control, early‐phase and late‐phase groups. (C) Uniform Manifold Approximation and Projection (UMAP) plots showing the *Il17a* gene expression of T cells across control and radiation groups in the early and late phases. (D) Volcano plot showing the differentially expressed genes between γδT cells and other T/NK sub‐clusters. (E) Representative flow cytometry plots for (F). (F) Quantification of IL17A^+^ cells of γδT cells in the lungs of mice during the late phase RP, early phase RP, and in control mice by flow cytometry. A two‐sided Wilcoxon test is adopted for comparing each group. ns, *p* ≥ .05, *p < .05, **p < .01, ***p < .001. (G) Violin plots showing the gene expression of interested genes across T/NK sub‐clusters. (H) The differences of IL17 response signature scores among sub‐clusters of major cell types. The signature scores are calculated by the AUCell algorithm (see Methods). A two‐sided Wilcoxon test is adopted for comparing one sub‐clusters and all other clusters. *p = 10E−10–200, **p = 10E−200–300, ***p < 10E−300. (I) UMAP plots showing the IL17 response signature scores. (J) The differences in IL17 response signature scores between the late‐phase group and the early‐phase group. The horizontal line denotes the average expression. The signature scores are calculated by the AUCell algorithm (see Methods). A two‐sided Wilcoxon test is adopted for comparing the early‐phase group and late‐phase group. *p = 10E−10–200, **p = 10E−200–300 and ***p < 10E−300.

We systematically analyzed Vγ6^+^Vδ4^+^ γδ17 T cells in RP, focusing on markers related to exhaustion (*Pdcd1*, *Havcr2*), activation (*Il1r1*, *Il23r* and *Il17re*), and so on. These γδ17 T cells showed a terminally exhausted phenotype, evident from *Tox* gene expression and absence of *Tcf7*, underlining their critical effector role and progression into a terminally exhausted state. Marked by *Itgae*, a tissue‐resident memory T cell indicator,^8^ γδ17 T cells likely expand from tissue‐resident memory cells rather than peripheral recruitment. Furthermore, the elevated expression of activation markers such as *Il1r1*, *Il23r* and *Il17re*, along with previous findings that *Il1β* (the ligand of *Il1r1*) is mainly expressed in neutrophils, suggests that γδ17 T cells are likely activated by IL1β released from neutrophils (Figure 3G and Figure S3C,D).

The IL17 response signature, a well‐established Gene Ontology term, was utilized to identify downstream targets of γδ17 T cells. Neutrophils exhibited the highest IL17 response signature activity (Figure 3H,I). Notably, γδ17 T cells were predominantly found in the late stage of RP, suggesting that IL17 response is limited to this phase. We then compared IL17 response signature scores between early and late‐stage neutrophils, finding significant enrichment in the late phase (Figure 3J). These findings indicate that, during late‐stage RP, tissue‐resident γδ17 T cells, activated by IL1β from neutrophils, expand and produce IL17A, thereby enhancing neutrophil recruitment to the lungs. Given that γδ17 T cells and neutrophils conspire to promote breast cancer metastasis,^9^ targeting γδ17 T/neutrophil axis may enhance efficacy while reducing toxicity. Specifically, we observed an upregulation of *Il17re* in γδ17 T cells, which is the receptor of *Il17c*. However, we did not detect any transcriptomic expression of *Il17c*, making it challenging to trace the origin of *Il17c*. Given previous reports suggesting that *Il17c* is upregulated earlier than *Il17a* in various inflammatory conditions,^10^ future studies should aim to detect *Il17c* expression at early time points.

In summary, our study provided a comprehensive and dynamic single‐cell immunological profile of RP progression from the early phase to the late phase (Figure 4). We also detail the roles of other cell types like macrophages, epithelial, and endothelial cells in RP response in Supporting Information (Figure S4‐S6). Briefly, we observed an early‐phase manifestation of increased oxidative stress response in pulmonary alveolar type II cells and compromised endothelial cell integrity, followed by a late‐phase presence of pro‐inflammatory Ly6c^hi^ macrophages in RP. These insights could illuminate RP pathogenesis and suggest new therapeutic avenues.

**FIGURE 4:**
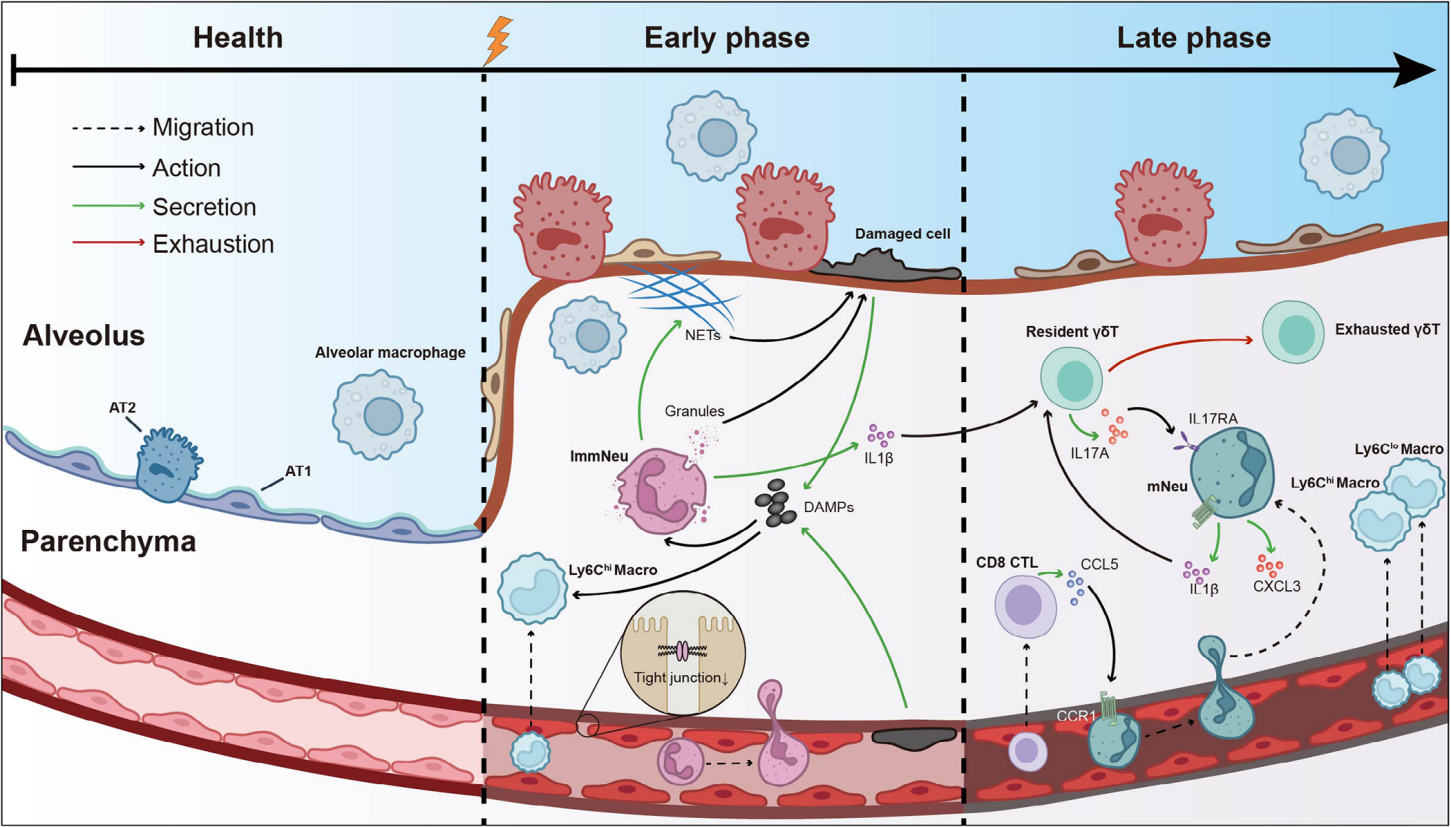
Summary of the biological process of radiation pneumonitis (RP) in early and late phases. In the early phase, radiation causes cell death and DAMPs release of epithelial and endothelial cells, which recruits neutrophils and macrophages. The immature neutrophils are granules‐rich and produce a lot of reactive oxygen species (ROS) and neutrophil extracellular traps (NETs), inducing more severe damage to lung tissue. Besides, some tight junction proteins are downregulated in endothelial cells and provide a preferential condition for immune infiltration. Notably, immature neutrophils secrete interleukin (IL)1β, which subsequently activates tissue‐resident γδ17T cells and mature neutrophils maintain this process. Reciprocally, γδ17T expand to produce IL17A to enhance the recruitment of neutrophils to lung tissue. CD8+T may also contribute to neutrophil recruitment through the CCL5–CCR1 axis.

## AUTHOR CONTRIBUTIONS

Conceptualization, methodology and project administration: Wenting Ren and Yibo Gao; Data curation: Wenting Ren; Formal analysis and visualization: Xiaoxiang Zhou, Ziming Jiang and Shiqi Li; Investigation and resources: Haoxuan Zhang; Writing‐original draft: Wenting Ren, Xiaoxiang Zhou, Ziming Jiang and Shiqi Li; Writing‐review & editing: Yibo Gao; Supervision: Jianrong Dai, Yexiong Li, Nan Bi, Yibo Gao and Jie He; Funding acquisition: Yibo Gao and Jie He; All authors read the manuscript, offered feedback and approved it before submission.

## CONFLICT OF INTEREST STATEMENT

The authors declare no conflict of interest.

## FUNDING INFORMATION

This work was supported by grants from the National Key R&D Program of China (2021YFC2501900), National Natural Science Foundation of China (82122053 and 82188102), R&D Program of Beijing Municipal Education Commission (KJZD20191002302), CAMS Initiative for Innovative Medicine (2021‐1‐I2M‐012), Key‐Area Research and Development Program of Guangdong Province (2021B0101420005), Shenzhen Science and Technology Program (RCJC20221008092811025 and ZDSYS20220606101604009), Shenzhen High‐level Hospital Construction Fund, Sanming Project of Medicine in Shenzhen [grant number SZSM202211011], Shenzhen Clinical Research Center for Cancer [grant number (2021)287], and Aiyou Foundation [grant number KY201701].

## ETHICS STATEMENT

All animal experiments conducted in this study were approved by the Ethics Committee of the Cancer Hospital Chinese Academy of Medical Sciences (Approval No. NCC2022A028).

## Supporting information

Supporting Information

Supporting Information

Supporting Information

## Data Availability

Raw and processed scRNA‐seq data, bulk RNA‐seq data, and proteomics data generated in this study can be obtained from Gene Expression Omnibus (GEO) with an accession number of GSE236049. All the data and code in this manuscript are accessible upon reasonable request.
