# Supplementary material for: Multitemporal single‐cell profiling decoding crosstalk between γδ17 T cells and neutrophils in radiation pneumonitis
Source: Clin Transl Med. 2024 May 17;14(5):e1700. doi: 10.1002/ctm2.1700 (PMC11101667; doi:10.1002/ctm2.1700)
Supplement: Supplementary file 1 — Supporting Information [file CTM2-14-e1700-s003.docx]

**Supplementary Figures**


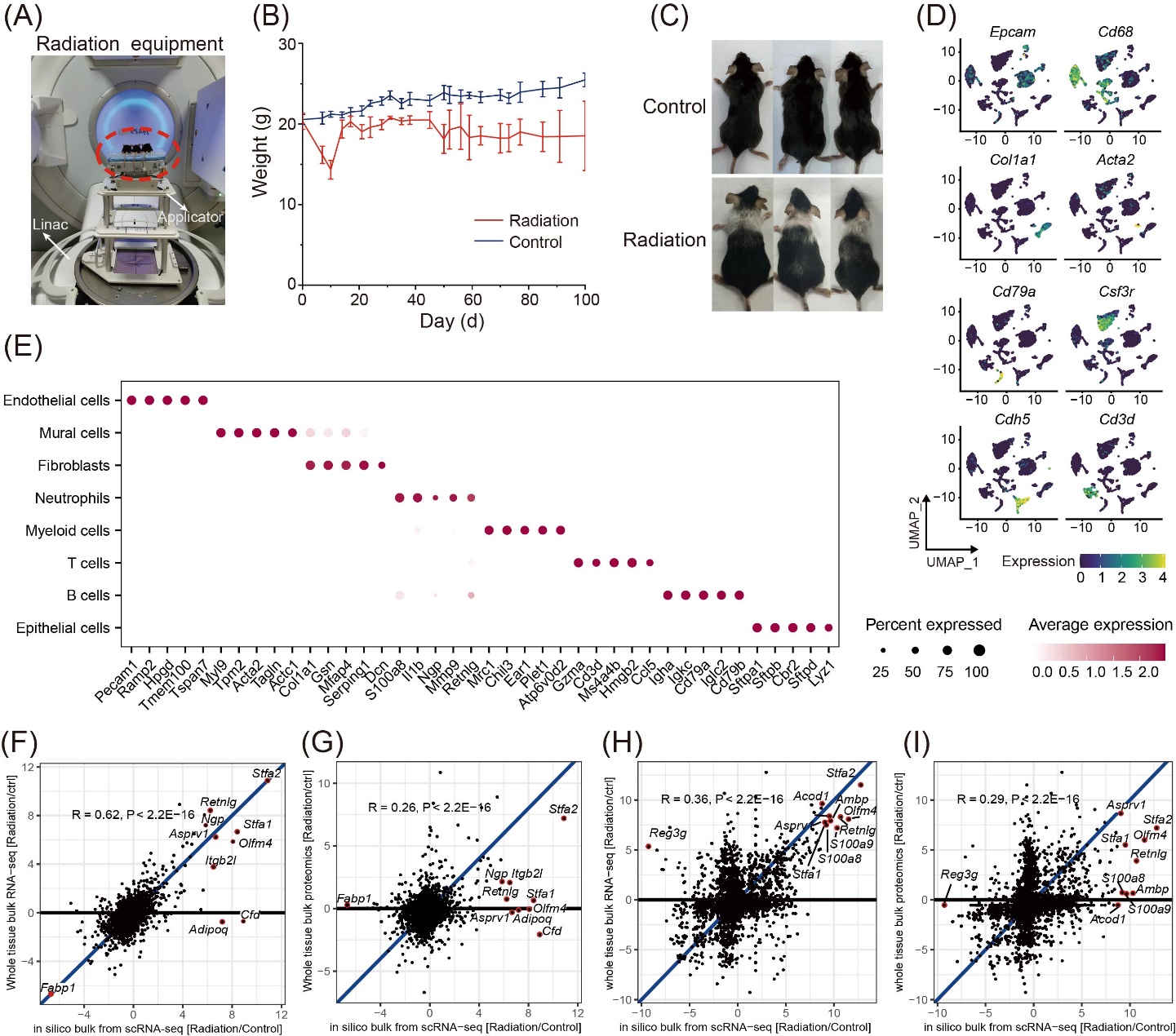


**Figure S1. Phenotypic changes of irradiated mice and identification of cell subtypes in radiation pneumonitis. (A)** Radiation equipment. Red circle indicates the placement, and white arrows indicate accessories. **(B)** Weight changes of mice (n = 3–7). **(C)** Hair color in the radiation site turned white in the late phase of RP. **(D,E)** Expression patterns of canonical markers and DEGs of each cell type. **(F–I)** Scatter plot of fold changes calculated between control and radiation groups for the bulk RNA-seq (**E, G**) and proteomics (**F, H**) (y-axis) with in silico bulk (x-axis) RNA-seq samples, **E** and **F** are the changes in the early phase of RP, **G** and **H** are the changes in the late phase of RP. The blue line represents Deming regression line. Red dots are the top 10 genes with the highest absolute average fold change across both modalities. Statistical significance was determined by Pearson correlation (p < 2.2e−16). RP, radiation pneumonitis; DEG, differentially expressed gene.

**
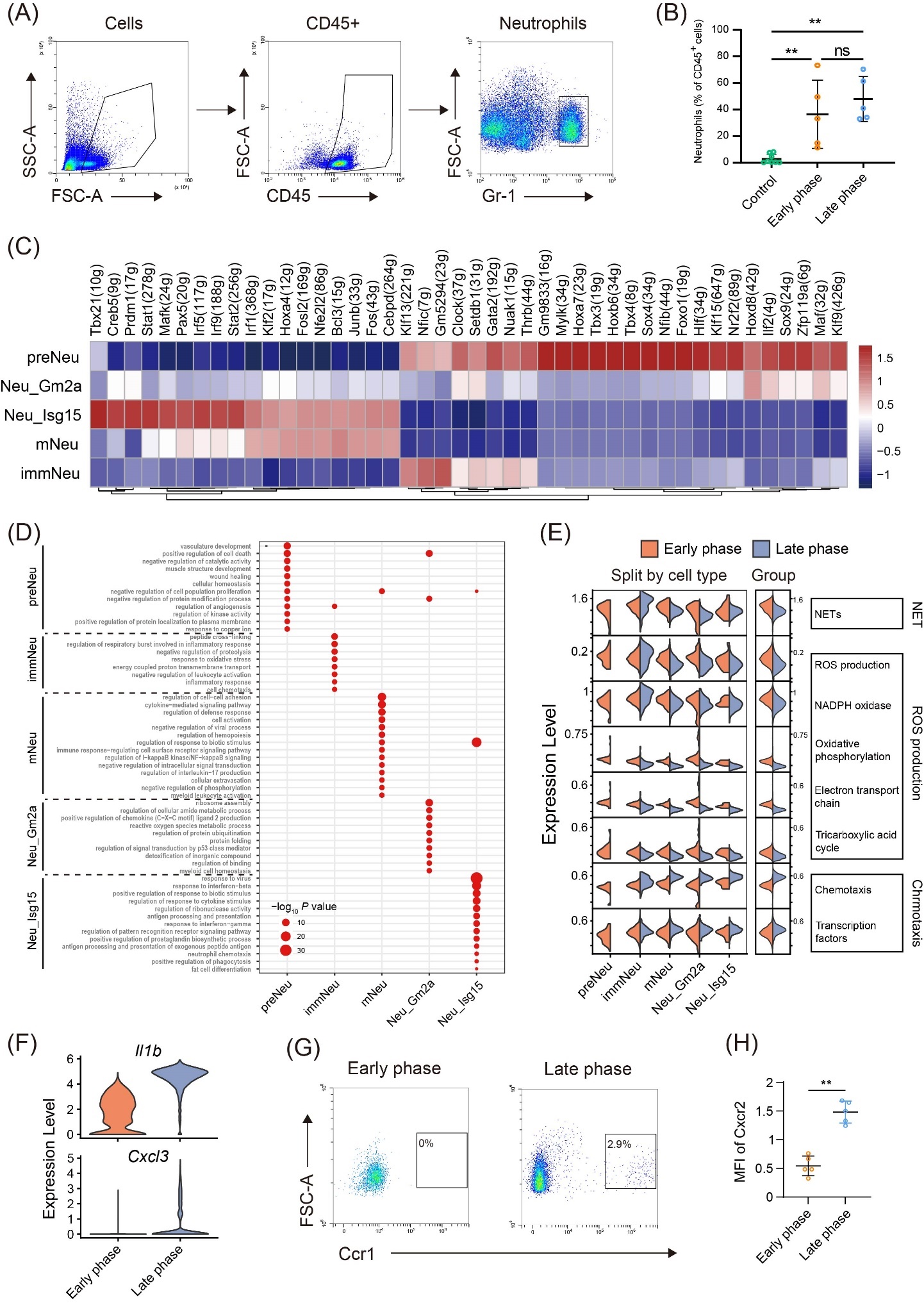
**

**Figure S2 Characteristics of neutrophil subsets in different phases of RP. (A)** Gating strategy of neutrophils. **(B)** Quantification of neutrophils of CD45^+^ cells in the lungs of mice during the late phase RP, early phase RP, and in control mice by flow cytometry (n = 7 control mice, n = 5 irradiated mice at early and late phase). A two-sided Wilcoxon test is adopted for comparing each group. ns, p ≥ 0.05, *p < 0.05, **p < 0.01, ***p < 0.001. **(C)** Transcription factors inferred by SCENIC. The number of target genes for each transcription factor is indicated in parentheses. **(D)** GO enrichment analysis of differential genes in each neutrophil subtype, Benjamini–Hochberg-corrected *P* values are analyzed by Fisher’s exact test, one-sided. **(E)** Violin plots showing the comparison of other selected pathway scores for the early and late phase group. **(F)** Violin plots showing expression of genes in neutrophils for early (orange) and late (blue) phase group. **(G)** Representative flow cytometry plots for (Fig. 2k). **(H)** MFI of Cxcr2 in the lungs of mice during the late phase RP and early phase RP by flow cytometry (n = 5 irradiated mice at early and late phase). ROS, reactive oxygen species.

**
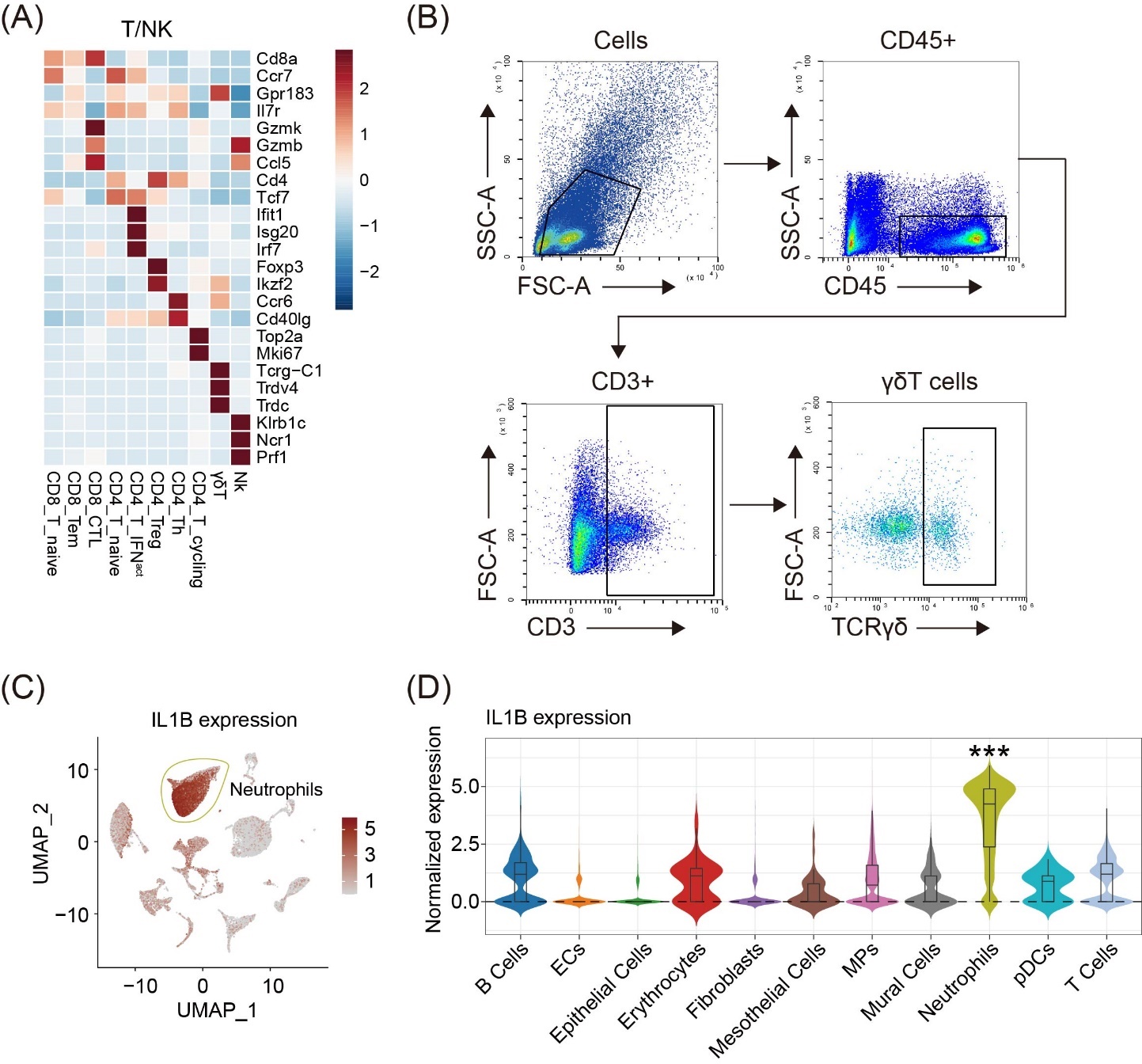
**

**Figure S3. Canonical markers of T/NK sub-clusters, gating strategy, and *Il1b* expression in major cell types. (A)** Heatmap showing the canonical marker genes of T/NK sub-clusters. **(B)** Gating strategy of γδT cells. (**C**) UMAP plots showing the *Il1b* expression across major cell types. (**D**) The differences of *Il1b* expression among major cell types.

**
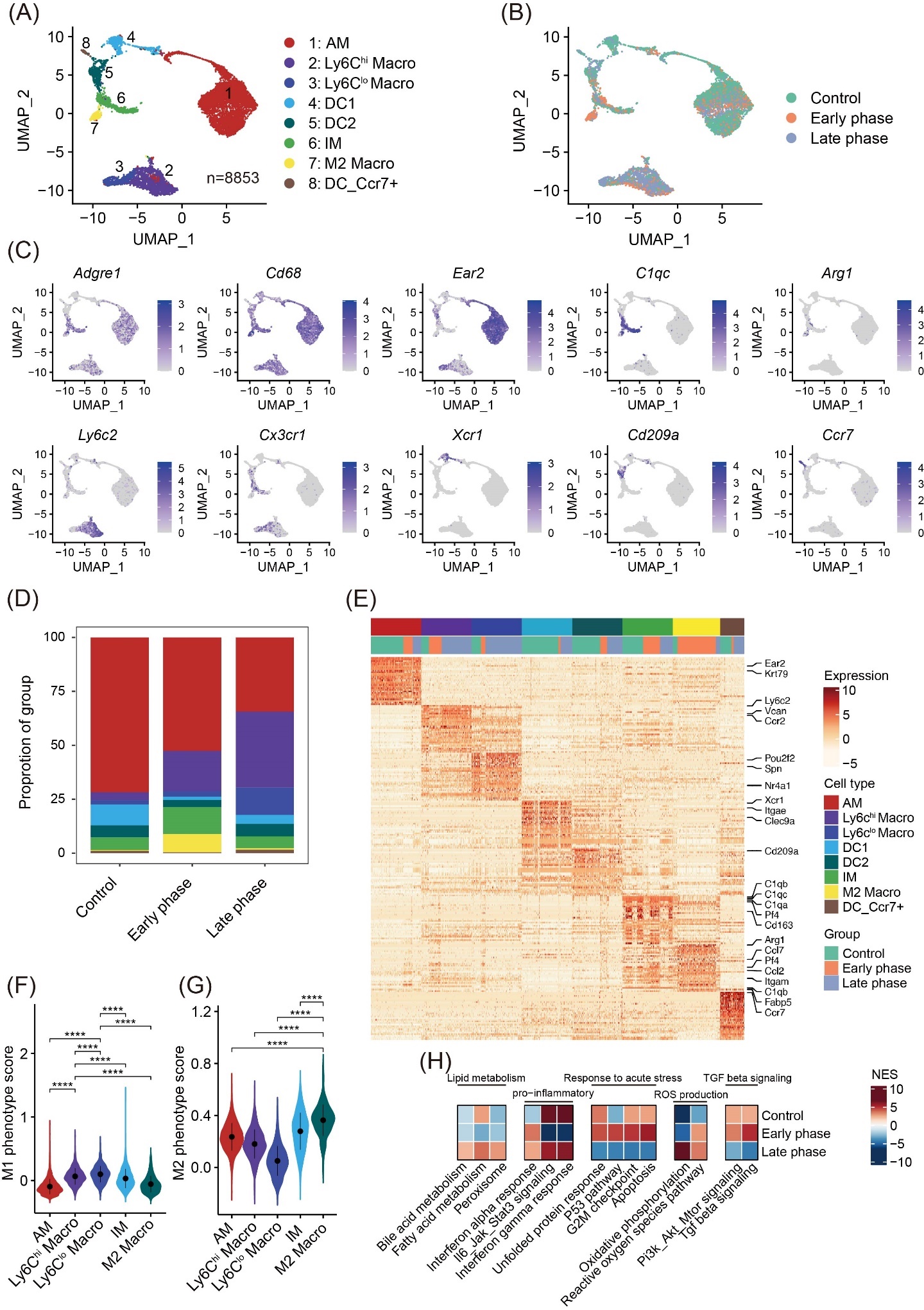
**

**Figure S4 Radiation-primed macrophages boost the whole inflammation process. (A)** Louvain clustering of 8853 myeloid cells into 8 subpopulations. **(B)** UMAP plot of myeloid cells, colored by different time points. **(C)** UMAP plots showing expression of selected genes in myeloid cell clusters. **(D)** Proportion of eight myeloid clusters in the three groups **(E)** Heatmap of transcriptional landscape of myeloid clusters. **(F, G)** Violin plots showing comparison of M1 **(F)** and M2 phenotype **(G)** score among macrophage subpopulations, two-sided Wilcoxon test. ****, p < 0.0001. (H) Heatmap showing the normalized enrichment score (NES) of indicated GO terms.

**
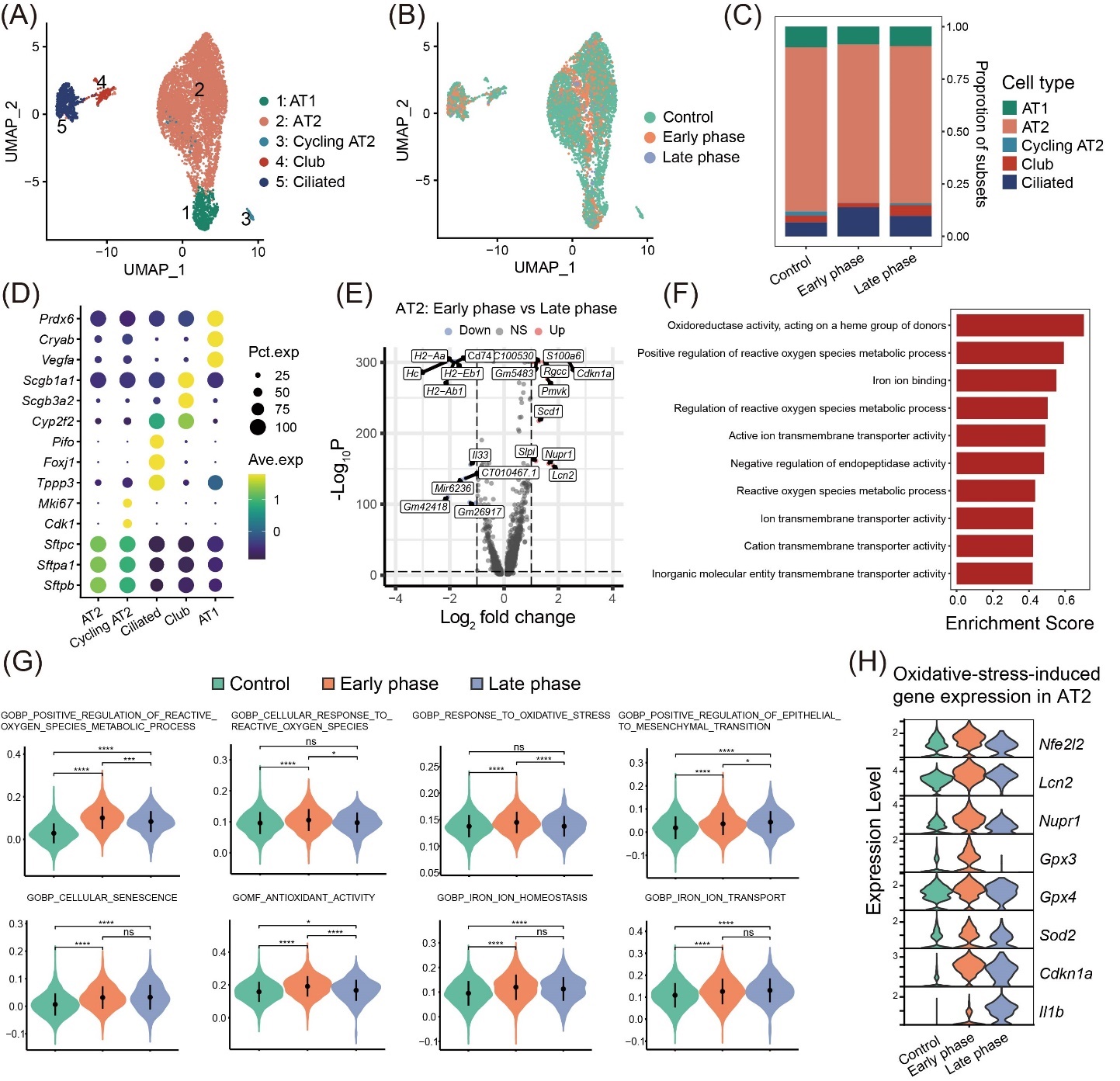
**

**Figure S5 Elevated oxidative stress of AT2 population in early phase of RP. (A)** Louvain clustering of epithelial cells into 5 subsets. **(B)** UMAP plot of epithelial cells, colored by different time points. **(C)** Cell ration of epithelial cells in each group. **(D)** Marker genes of epithelial subpopulations. **(E)** Top 10 differentially expressed genes of AT2 in the early phase vs control group. **(F)** GSEA pathway of AT2 in the early phase vs control group. **(G)** Violin plots showing comparison of GO term signature among AT2, two-sided Wilcoxon test, ****, p < 0.0001. **(H)** Violin plots showing gene expression related with oxidative stress and cell senescence.

**
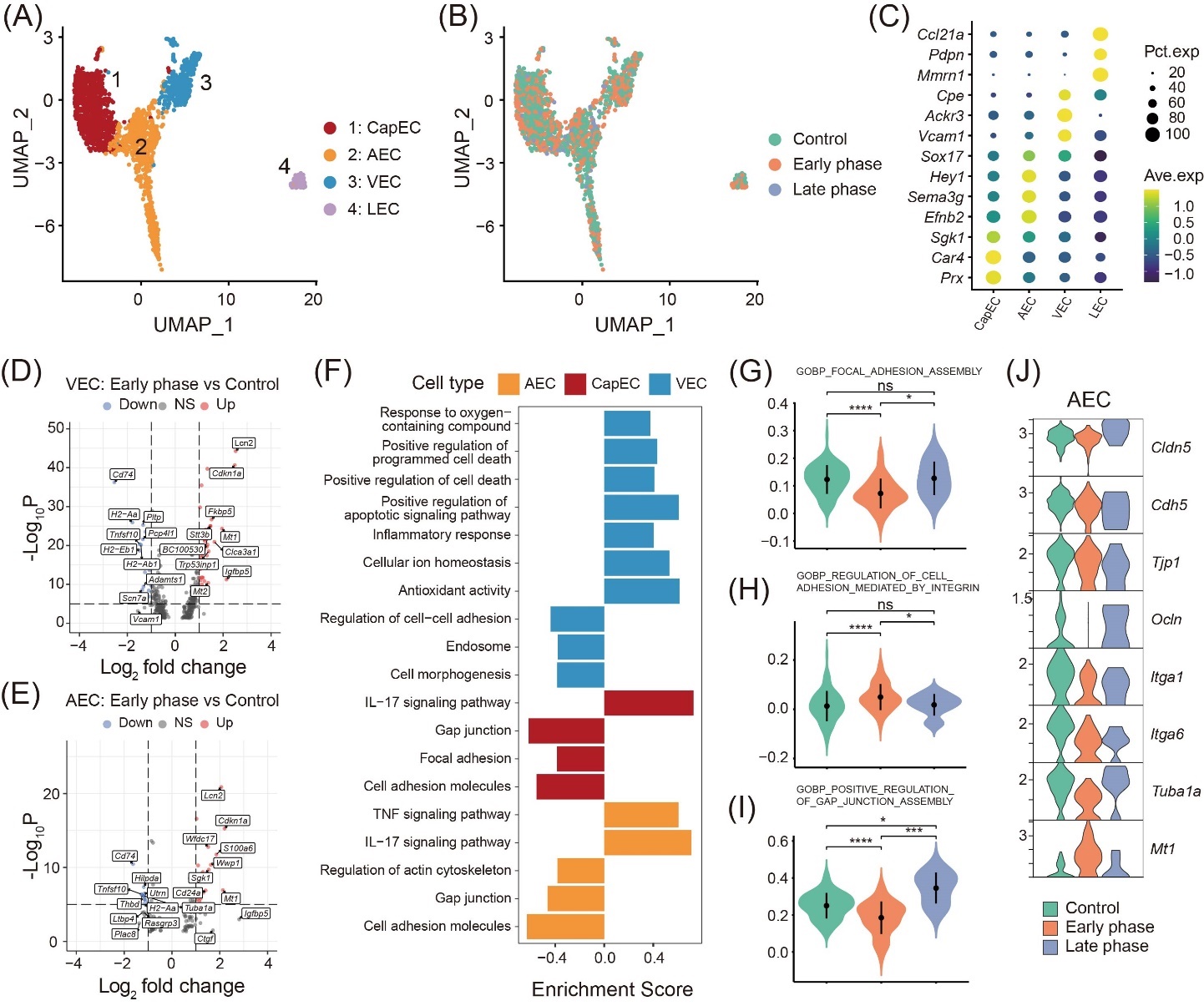
**

**Figure S6 Expression profile of impaired endothelial integrity after radiation.** **(A)** Louvain clustering of endothelial cells into 4 subpopulations. **(B)** UMAP plot of epithelial cells, colored by different time points. **(C)** Marker genes of endothelial subpopulations. **(D)** Top 10 differentially expressed genes of VEC in the early phase vs control group. **(E)** Top 10 differentially expressed genes of AEC in the early phase vs control group. **(F)** GSEA pathway of different subsets of endothelial cells in the early phase and the late phase group. **(G, H)** Violin plots showing adhesion assembly and gap junction assembly signature in AEC, two-sided Wilcoxon test, ****, p < 0.0001. **(I)** Dot plots showing comparison of oxidative stress and inflammation related genes.
