# Supplementary material for: Multitemporal single‐cell profiling decoding crosstalk between γδ17 T cells and neutrophils in radiation pneumonitis
Source: Clin Transl Med. 2024 May 17;14(5):e1700. doi: 10.1002/ctm2.1700 (PMC11101667; doi:10.1002/ctm2.1700)
Supplement: Supplementary file 2 — Supporting Information [file CTM2-14-e1700-s002.docx]

**Supplementary Tables**

Table S1 Quality control of scRNA-seq

|  | Control (day 0) | Early phase (day 10) | Late phase (day 100) |
| --- | --- | --- | --- |
| All cells | 17431 | 11059 | 11777 |
| Low quality cells (nFeature_RNA < 200 and > 8000; nCount_RNA < 200; percent.mt > 10%) | 106 | 34 | 69 |
| Doublets | 5758 | 4031 | 2116 |
| Remaining Cells | 11567 | 6994 | 9592 |
