# Supplementary material for: Multitemporal single‐cell profiling decoding crosstalk between γδ17 T cells and neutrophils in radiation pneumonitis
Source: Clin Transl Med. 2024 May 17;14(5):e1700. doi: 10.1002/ctm2.1700 (PMC11101667; doi:10.1002/ctm2.1700)
Supplement: Supplementary file 3 — Supporting Information [file CTM2-14-e1700-s001.docx]

Supplementary Materials for

**Multi-temporal single-cell profiling decodes cross-talk of γδ17 T cells and neutrophils in radiation pneumonitis**

Wenting Ren†, Xiaoxiang Zhou†, Ziming Jiang†, Shiqi Li†, Haoxuan Zhang, Jianrong Dai, Yexiong Li, Nan Bi, Yibo Gao✉, Jie He✉

These authors contributed equally: Wenting Ren, Xiaoxiang Zhou, Ziming Jiang, Shiqi Li

Correspondence to: Yibo Gao ([gaoyibo@cicams.ac.cn](mailto:gaoyibo@cicams.ac.cn)), Jie He ([hejie@cicams.ac.cn](mailto:hejie@cicams.ac.cn))

**This PDF file includes:**

Supplementary Results

Supplementary Discussion

Supplementary Materials and Methods

**Supplementary Results**

**Ly6c^hi^ macrophages promote chronic inflammation in the late phase of RP**

To gain insights into the molecular mechanisms of myeloid cells in RP, we categorized myeloid cells into eight subsets, including five macrophage types [alveolar macrophages (*Ear2*^+^, AM), interstitial macrophages (*C1qc*^+^, IM), M2 macrophages (*Arg1*^+^), Ly6c^hi^ macrophages (*Ly6c2*^+^), and Ly6c^lo macrophages (*Cx3cr1*^+^)] and three dendritic cell types [DC1 (*Xcr1*^+^), DC2 (*Cd209a*^+^), and *Ccr7*^+^ DC (*Ccr7*^+^)] (Figure S4A–S4C). We noted significant increases in M2 macrophages during the early phase and Ly6c^lo^ macrophages in the late phase, with Ly6c^hi^ macrophages rising in both phases (Figure S4D). Radiation exposure is known to drive monocyte recruitment and differentiation into macrophages, including Ly6c^hi^ and Ly6c^lo^ types^1-4^. Despite the contentious role of macrophages in radiation-induced inflammation, their distinct transcriptomic profiles are evident in a heatmap (Figure S4E), illustrating myeloid cell diversity in RP.

Classically activated (M1) macrophages fuel inflammation, while alternatively activated (M2) macrophages facilitate tissue repair and dampen inflammation ^5-7^. Assessing myeloid cell function through M1 and M2 signatures^8^, we found Ly6c^hi^ and Ly6c^lo^ macrophages predominantly exhibit a proinflammatory M1 phenotype (Figure S4F), whereas M2 macrophages leaned towards an anti-inflammatory M2 phenotype (Figure S4G). Notably, while the early phase saw an upsurge in anti-inflammatory M2 macrophages, the late phase was marked by an increase in proinflammatory M1-like macrophages, including Ly6c^hi^ macrophages and ly6c^lo^ macrophages, which suggests a macrophage transition from anti-inflammatory to proinflammatory states during RP progression.

We further focused on the functional changes in Ly6c^hi^ macrophages, which were enriched in both the early and late phases. According to the GSEA results of the Ly6c^hi^ macrophage subpopulations, proinflammatory signaling pathways, lipid metabolism, and ROS production pathways were enriched in the late phase (Figure S4H), suggesting that Ly6c^hi^ macrophages were actively involved in chronic inflammation. Conversely, in the early phase, pathways associated with the stress response and TGFβ were enriched (Figure S4H). TGFβ can activate *Arg1* expression in macrophages in an autocrine or paracrine manner to upregulate the M2 gene profile and restrict inflammation^9^. Based on these findings, we hypothesized that Ly6c^hi^ macrophages in the early phase enhanced the anti-inflammatory function of M2 (*Arg1*^+^) macrophages through the TGFβ pathway. In summary, macrophage function changed in response to radiation exposure, and Ly6c^hi^ macrophages could enhance anti-inflammatory functions in the early stage through the TGFβ pathway and proinflammatory activity in the late phase.

**Radiation induces oxidative stress-related gene expression in AT2 cells in the early phase of RP**

In our study, we classified 6,620 epithelial cells into five subsets [pulmonary alveolar type I cells (*Vegfa^+^*, AT1), pulmonary alveolar type II cells (*Sftpc^+^*, AT2), cycling AT2 (*Mki67^+^*), ciliated cells (*Foxj1^+^*), and club cells (*Scgb1a1^+^*)] based on their expression profiles^10^ (Figure S5A, 5D). We observed a predominance of AT2 cells over other epithelial subsets (Figure S5A–S5C). Known as alveolar stem cells, AT2 cells are critical for alveolar homeostasis and repair post-injury^11^. While previous studies have noted senescence and inflammation in RP-affected AT2 cells^12^, the specific impact of radiation on AT2 cells during the early and late phases of RP has not been fully explored. In our analysis, we noted a significant upregulation of antioxidant genes such as *Lcn2* and *Nupr1* in both phases (Figure S5E), and found an enrichment of oxidative stress response and iron ion binding pathways in AT2 cells through Gene Set Enrichment Analysis (GSEA) (Figure S5F). *Lcn2*, a direct target of Nupr1, is implicated in ferroptosis—a unique oxidative metabolic process. The upregulation of Nupr1 and Lcn2 suggests their role in mitigating ferroptosis, reducing oxidative stress, and decreasing cell death^13^. Therefore, we hypothesized that ferroptosis could participate in initial damage and death in AT2 cells as a unique form of oxidative metabolic process.

Considering the role of reactive oxygen species (ROS) as toxic byproducts of radiation contributing to lung injury, we investigated the long-term effects of radiation on epithelial cells, focusing on oxidative stress response and ROS pathways. Our findings indicate that AT2 cells in the early phase of RP exhibit a higher oxidative response signature compared to the late phase, suggesting that radiation-induced damage is primarily confined to the early phase (Figure S5G). Except for *Lcn2* and *Nupr1*, several other antioxidant genes like *Nfe2l2*, *Gpx3*, *Gpx4*, and *Sod2*^14-16^ were upregulated in the early phase (Figure S5H). Interestingly, the signatures of iron ion homeostasis, senescence, and epithelial-to-mesenchymal transition remained elevated in the late phase of RP, highlighting the persistent disturbed state of AT2 cells (Figure S5G).

**Disrupting endothelial integrity permits inflammation after radiation in the early phase of RP**

We identified four distinct endothelial subsets [arterial endothelial cells (*Sema3g^+^*, AEC), venous endothelial cells (*Vcam1^+^*, VEC), capillary endothelial cells (*Prx^+^*, CapEC), and lymphatic endothelial cells (*Mmrn1^+^*, LEC)] (Figure S6A–S6C). Radiation-induced modulation of endothelial cell adhesion molecules, such as PECAM-1, VE-cadherin, and Claudin5, was previously shown to be reduced in vitro^17^. However, the heterogeneous function of endothelial subsets in irradiated lungs remains to be investigated.

To investigate functional alterations in vascular endothelial cells in the early phase of RP, we performed GSEA. Several pathways that are induced by cellular stress or inflammation were enriched in irradiated VECs (Figure S6F). Additionally, we observed an increase in Igfbp5, linked to inflammatory lung injury^18^, and Cdkn1a, associated with oxidative stress responses^19^, in the early phase of RP (Figure S6D, S6E).

In contrast to VECs, we noted a decrease in gap junction and cell adhesion molecules in AECs and CapECs during the early phase of RP (Figure S6F), indicating compromised endothelial barrier integrity. AECs are known to express genes for tight and gap junction proteins, crucial for wall strength and elasticity^10^. Evaluating cell adhesion and gap junction assembly in AECs, we found lower scores in the early phase of RP compared to the late phase and control (Figure S6G, S6H), suggesting early-phase impairment in maintaining endothelial integrity, which seems to recover later. Several essential genes involved in gap and tight junctions (*Tjp1*, *Cdh5*, *Cldn5*, and *Ocln*) and anchoring junctions (*Itga1*, *Itga6*, and *Tuba1a*) were downregulated in AECs (Figure S6I), associated with endothelial leakiness and alveolar exudate post-radiation. Additionally, Mt1, a transmembrane metalloprotease (MT1-MMP), was upregulated in AECs during the early phase of RP (Figure S6E, S6I). Mt1 functions as a transmembrane metalloprotease in the form of membrane type 1-matrix metalloproteinase (MT1–MMP), contributing to the degradation of interstitial collagens and extracellular matrix, further impairing endothelial integrity and leading to alveolar flooding and immune infiltration.

**Supplementary Discussion**

This study had several limitations. Firstly, this study primarily focused on murine models and lacked human data, which may limit its direct translatability to human RP. Secondly, while the study identified some promising potential therapeutic targets, the investigation of the effect of clinical intervention on improving RP is lacking. Thirdly, the murine models used in this study did not have lung cancer, which may impact the immune microenvironment of lung tissue. Therefore, the results should be interpreted with caution.

**Supplementary Materials and methods**

**Irradiation of the animal models.** All animal experiments conducted in this study were approved by the Ethics Committee of the Cancer Hospital Chinese Academy of Medical Sciences. Eight to nine-week-old C57BL/6N female mice were purchased from Beijing Vital River Laboratory Animal Technology Co. Ltd. (Beijing, China). All of the mice were maintained in a specific pathogen-free environment with a constant temperature of 20–26°C. The relative humidity is 40%–70% and a regular light interval is 12h/12h at the Laboratory Animal Center, Beijing Brightshines Technology Co., Ltd. The laboratory animal requirements of the environment and housing facilities meet the relevant requirements of the national standard GB14925-2010.

Irradiation was carried out using an Elekta clinical accelerator at a dose rate of 0.1Gy/s. A 20 Gy dose of 9 MeV electron-ray was administered through a 15cm×15cm applicator with a custom lead block, followed by a 1 cm bolus, targeting the mice's thorax. A specially designed lead block with a 2.5cm × 11cm opening ensured thorax-specific irradiation. The process was conducted under anesthesia (tribromoethanol, 250 mg/kg intraperitoneally), treating two or three mice simultaneously. Dosimetry for each mouse involved GafchromicTM EBT-XD films (Ashland Inc., Bridgewater NJ, USA) placed in front of their chest for immediate optical density (OD) measurements post-RT. The films were then scanned 48 hours later using an Epson scanner (https://epson.com/scanners) and analyzed with RIT software (https://radimage.com) for precise dosing.

**Pulmonary function test.** All mice were placed in the Buxco non-invasive airway mechanics (NAM) plethysmograph (Data Sciences International [DSI] St. Paul, MN). Over a period of 10 minutes, mice were monitored and different ventilatory parameters were determined by the finepointe software: frequency (f), tidal volume (TV), minute volume (MV), peak inspiratory flows (PIF), peak expiratory flows (PEF), time of inspiration (Ti), time of expiration (Te), midexpiratory flow (EF50).

**Lung isolation and Tissue dissociation.** The mice (n=3–7 per group) were sacrificed by cervical dislocation and lungs were isolated at 10 and 100 days post-radiation for scRNAseq, bulk RNAseq, proteomics analysis, and H&E staining. Several pieces of lungs were installation-fixed for 5 min at 20 cm H_2_O hydrostatic pressure with 1.5% (w/v) paraformaldehyde (PFA) (Sigma- Aldrich, Oakville, ON, Canada) and 1.5% (w/v) glutaraldehyde (Sigma-Aldrich, Oakville, ON, Canada) in 150 mM HEPES (Sigma-Aldrich, Oakville, ON, Canada). After isolation, lungs were kept in this solution for 48 hours at 4°C before paraffin embedding. Paraffin-embedded sections (4µm thick) were then stained with H&E. This process included tissue dehydration, embedding, sectioning, and staining.

Fresh tissues were preserved in sCelLiveTM Tissue Preservation Solution (Singleron Bio Com, Nanjing, China) on ice within 30 minutes post-surgery. Specimens were washed thrice with Hanks Balanced Salt Solution (HBSS) 3 times and then digested with 2 ml sCelLiveTM Tissue Dissociation Solution (Singleron) by Singleron PythoN™ Automated Tissue Dissociation System (Singleron) at 37 ℃ for 15 mins. Afterwards, the GEXSCOPE® red blood cell lysis buﬀer (Singleron, 2ml) was added, and cells were incubated at 25°C for another 10 mins to remove red blood cells. After centrifugation at 500 × g for 5 minutes, cells were resuspended in PBS and stained with trypan blue (Sigma, United States) to assess viability microscopically.

**Library preparation and scRNA-seq data processing.** For library construction, lung tissues from three mice in each group were pooled. Single-cell suspensions (1×10^5^ cells/ml) with PBS (HyClone) were loaded into microfluidic devices using the Singleron Matrix® Single Cell Processing System (Singleron). Subsequently, the scRNA-seq libraries were constructed according to the protocol of the GEXSCOPE® Single Cell RNA Library Kits (Singleron)^20^. Individual libraries were diluted to 4 nM and pooled for sequencing. At last, pools were sequenced on Illumina NovaSeq6000 with 150 bp paired-end reads.

Raw reads were processed to generate gene expression profiles using an internal pipeline. Briefly, cell barcode and UMI were extracted after filtering read one without poly T tails. Adapters and poly A tails were trimmed before aligning read two to GRCm38 with ensemble version 92 gene annotation (fastp 2.5.3a and feature Counts 1.6.2)^21^. Reads with the same cell barcode, UMI, and gene were integrated together in each cell.

**Quality control and batch correction.** To filter out low-quality cells and doublets, the cells that had either fewer than 200 unique molecular identifiers (UMIs), or over 8000 or below 200 expressed genes, were removed. Cells that had over 10% UMIs derived from the mitochondrial genome were further removed. Table S1 presents the results of quality control.

To merge samples across the tissues, we run a canonical correlation analysis (CCA) for batch correction using the Run-MultiCCA function in the R package Seurat (v3.1.2)^22^. Then we performed Uniform Manifold Approximation and Projection (UMAP) dimensionality reduction using the RunUMAP function in Seurat.

**Clustering and Differential expression analysis.** For cell clustering, we utilized Seurat's FindClusters function and identified genes differentiating these clusters using the Wilcoxon rank-sum test via Seurat's FindAllMarkers function^22^. Each major cluster was then isolated and processed using the same normalization and integrated approach. Initial clustering at a subset-specific resolution was followed by the removal of rare clusters containing cells with markers of contaminant cell types (e.g., a *Pecam1*^+^ cluster in the epithelial subset), likely representing rare doublets missed in demultiplexing. After excluding contaminants, we re-clustered the integrated data to identify distinct cell types within each subset, based on known expression signatures.

Gene enrichment analysis was conducted using gene oncology enrichment and GSEA. To measure neutrophil maturation, we employed the "AddModuleScore" function from the Seurat package (version 3.1.2) with default settings to calculate signature scores^23^. To assess the activity of specific gene signatures, we employed the AUCell package, calculating signature scores for each single cell^24^.

**Construction of Intercellular Communication Networks.** Chemokine receptor-ligand pairs were evaluated with CellPhoneDB (version 2.1.1)^25^, based on a curated chemokine receptor-ligand interaction map previously described^26^. We utilized dotplot function to visualize the potential interaction strength between ligand and receptor, which was predicted based on their average expression. Significant ligand–receptor pairs (p < 0.05) were extracted for illustration.

**Trajectory analysis.** Monocle3 (v1.2.7) algorithm was utilized to investigate interconversion and evolutionary trajectories of different cell types. Signature genes expressed in at least 10% cells of the dataset and with a p < 0.01 calculated using the differentialGeneTest function were included to define the trajectory progress. Pseudotime-dependent genes were calculated using differentialGeneTest and the “fullModelFormulaStr” option “~sm.ns(Pseudotime)”.

**SCENIC analysis.** Transcription factor network was constructed by SCENIC^24^, using scRNA expression matrix and transcription factors in AnimalTFDB. Genes involved in the predicted regulatory network were defined as a gene set, and AUCell package calculated the AUC value of the gene set to assess the activity of regulatory network in cells.

**Whole tissue bulk RNA sequencing.** Total RNA was extracted from each sample of lung tissue (control group or irradiation group) using TRIzol reagent according to the manufacturer’s protocol (Invitrogen, California). The RNA was quantified using a NanoDrop spectrophotometer (Thermo, California). For RNA high-throughput sequencing, mRNA libraries were created from each group using the Hieff NGS MaxUp ll Dual-mode mRNA library prep kit from Illumina (San Diego, CA), and sequenced by DNBSEQ-T7 (GenePlus-Shenzhen, Shenzhen, China). Fastp software (https://github.com/OpenGene/fastp) is used to joint sequences, filter low-quality and N-base, and obtain high-quality clean data. For the analysis of differentially expressed genes, the clean data for each sample were aligned to the mus musculus reference genome (https://ftp.ncbi.nlm.nih.gov/genomes/refseq/vertebrate_mammalian/Mus_musculus/all_assembly_versions/GCF_000001635.27_GRCm39/GCF_000001635.27_GRCm39_genomic.fna.gz) using STAR (version 2.7.6a) software. We used StringTie2 software to quantify gene expression levels and standardized the expression levels using FPKM (Fragments per Kilobase Million) and TPM (Transcripts Per Million) methods.

**Proteomics analysis.** The samples were grinded with liquid nitrogen and then were added lysis buffer (1% Triton X-100, 1% protease inhibitor, 50 μM PR-619, 3 μM TSA, 50 mM NAM) for ultrasonic cracking. We utilized BCA kit to determine protein concentrations and then digested the protein into peptides with trypsin. Then we dissolved peptides in liquid chromatography mobile phase A and separated them with EASY-nLC 1200 ultra-high performance liquid system. The separated peptides were detected by Orbitrap Exploris™ 480 mass spectrometry.

**Multi-omics data integration.** We combined bulk RNA-seq and proteomics data from the same batch of scRNA-seq to validate changes in global expression patterns seen at the single-cell level. All counts inside a mouse sample were added up to create the in silico bulk sample. Then the whole-lung tissue bulk data and the in silico bulk data were both standardized for log2 transformation. The in silico bulk, whole-lung tissue bulk RNA-seq, and proteomics data were merged on intersecting genes in three data sets.

**Multiplex immunofluorescence staining.** Multiplex immunohistochemistry (IHC) staining techniques were applied to identify neutrophils and NETs within mouse lung tissues. Neutrophils were detected through neutrophil elastase (NE) positive staining in conjunction with DAPI, while NETs were highlighted by co-staining for NE and citrullinated histone-H3 (Cit-H3). This multiplex immunofluorescence staining utilized the PANO 7-plex IHC kit (catalog #0004100100, Panovue, Beijing, China). The primary antibodies included rabbit anti-histone H3 (catalog #ab5103, 1:200, Abcam) and rabbit anti-mouse NE (catalog #BS-6982R, 1:100, Bioss), followed by incubation with horseradish peroxidase-conjugated secondary antibody and tyramide signal amplification (TSA). Slides were subjected to microwave treatment post each TSA step. Following the labeling of all human antigens, nuclei were stained with 4′,6-diamidino-2-phenylindole (DAPI, catalog #D9542, 1:2000, Sigma). Fluorescent images of whole slides were captured using an Olympus VS200 system (Olympus Germany) equipped with an Olympus UPLXAPO 20x objective lens. The analysis of whole slide fluorescence images was conducted using QuPath software.

**Flow cytometry.** For the identification of neutrophils, the antibodies used included Brilliant Violet 510™ anti-mouse CD45 (catalog #103138, BioLegend), Alexa Fluor® 594 anti-mouse Ly-6G/Ly-6C (Gr-1) (catalog #108448, BioLegend), APC/Cyanine7 anti-mouse CD184 (CXCR4) (catalog #146523, BioLegend), PE/Cyanine7 anti-mouse CD182 (CXCR2) (catalog #149315, BioLegend), and APC anti-mouse CD191 (CCR1) (catalog #152503, BioLegend). For T cells, the antibodies employed were APC anti-mouse CD45 (catalog #103112, BioLegend), Alexa Fluor® 700 anti-mouse CD3 (catalog #100216, BioLegend), APC/Cyanine7 anti-mouse TCR γ/δ (catalog #118143, BioLegend), and IL-17A, PE-Cyanine7 (catalog #25-7177-80, eBioscience). Stained cells were analyzed using a CytoFlex S flow cytometer (Beckman Coulter) and FlowJo software for data analysis.

**References**

1. Park H-R, Jo S-K, Jung U. Thoracic Irradiation Recruit M2 Macrophage into the Lung, Leading to Pneumonitis and Pulmonary Fibrosis. *J. Radiat. Prot. Res*. 2017;42(4):177-188.

2. Meziani L, Deutsch E, Mondini M. Macrophages in radiation injury: a new therapeutic target. *Oncoimmunology*. 2018;7(10):e1494488. doi:10.1080/2162402X.2018.1494488.

3. Groves AM, Johnston CJ, Williams JP, Finkelstein JN. Role of Infiltrating Monocytes in the Development of Radiation-Induced Pulmonary Fibrosis. *Radiat Res*. 2018;189(3):300-311. doi:10.1667/RR14874.1.

4. Groves AM, Johnston CJ, Misra RS, Williams JP, Finkelstein JN. Whole-Lung Irradiation Results in Pulmonary Macrophage Alterations that are Subpopulation and Strain Specific. *Radiat Res*. 2015;184(6):639-649. doi:10.1667/RR14178.1.

5. Wynn TA. Integrating mechanisms of pulmonary fibrosis. *J Exp Med*. 2011;208(7):1339-1350. doi:10.1084/jem.20110551.

6. Wynn TA, Ramalingam TR. Mechanisms of fibrosis: therapeutic translation for fibrotic disease. *Nat Med*. 2012;18(7):1028-1040. doi:10.1038/nm.2807.

7. Zhang H, Han G, Liu H, et al. The development of classically and alternatively activated macrophages has different effects on the varied stages of radiation-induced pulmonary injury in mice. *J Radiat Res*. 2011;52(6):717-726. doi:10.1269/jrr.11054.

8. Zhang L, Li Z, Skrzypczynska KM, et al. Single-Cell Analyses Inform Mechanisms of Myeloid-Targeted Therapies in Colon Cancer. *Cell*. 2020;181(2):442-459.e29. doi:10.1016/j.cell.2020.03.048.

9. Zhou X, Spittau B, Krieglstein K. TGFβ signalling plays an important role in IL4-induced alternative activation of microglia. *J Neuroinflammation*. 2012;9:210. doi:10.1186/1742-2094-9-210.

10. Schupp JC, Adams TS, Cosme C, Jr., et al. Integrated Single-Cell Atlas of Endothelial Cells of the Human Lung. *Circulation*. 2021;144(4):286-302. doi:10.1161/CIRCULATIONAHA.120.052318.

11. Olajuyin AM, Zhang X, Ji HL. Alveolar type 2 progenitor cells for lung injury repair. *Cell Death Discov*. 2019;5:63. doi:10.1038/s41420-019-0147-9.

12. Wu F, Zhang Z, Wang M, et al. Cellular Atlas of Senescent Lineages in Radiation- or Immunotherapy-Induced Lung Injury by Single-Cell RNA-Sequencing Analysis. *Int J Radiat Oncol Biol Phys*. 2023;doi:10.1016/j.ijrobp.2023.02.005.

13. Liu J, Song X, Kuang F, et al. NUPR1 is a critical repressor of ferroptosis. *Nat Commun*. 2021;12(1):647. doi:10.1038/s41467-021-20904-2.

14. Forcina GC, Pope L, Murray M, et al. Ferroptosis regulation by the NGLY1/NFE2L1 pathway. *Proc Natl Acad Sci U S A*. 2022;119(11):e2118646119. doi:10.1073/pnas.2118646119.

15. Dodson M, Castro-Portuguez R, Zhang DD. NRF2 plays a critical role in mitigating lipid peroxidation and ferroptosis. *Redox Biol*. 2019;23:101107. doi:10.1016/j.redox.2019.101107.

16. Alim I, Caulfield JT, Chen Y, et al. Selenium Drives a Transcriptional Adaptive Program to Block Ferroptosis and Treat Stroke. *Cell*. 2019;177(5):1262-1279 e25. doi:10.1016/j.cell.2019.03.032.

17. Kabacik S, Raj K. Ionising radiation increases permeability of endothelium through ADAM10-mediated cleavage of VE-cadherin. *Oncotarget*. 2017;8(47):82049-82063. doi:10.18632/oncotarget.18282.

18. Yasuoka H, Zhou Z, Pilewski JM, Oury TD, Choi AM, Feghali-Bostwick CA. Insulin-like growth factor-binding protein-5 induces pulmonary fibrosis and triggers mononuclear cellular infiltration. *Am J Pathol*. 2006;169(5):1633-42. doi:10.2353/ajpath.2006.060501.

19. Badie C, Dziwura S, Raffy C, et al. Aberrant CDKN1A transcriptional response associates with abnormal sensitivity to radiation treatment. *Br J Cancer*. 2008;98(11):1845-51. doi:10.1038/sj.bjc.6604381.

20. Dura B, Choi JY, Zhang K, et al. scFTD-seq: freeze-thaw lysis based, portable approach toward highly distributed single-cell 3' mRNA profiling. *Nucleic Acids Res*. 2019;47(3):e16. doi:10.1093/nar/gky1173.

21. Chen S, Zhou Y, Chen Y, Gu J. fastp: an ultra-fast all-in-one FASTQ preprocessor. *Bioinformatics*. 2018;34(17):i884-i890. doi:10.1093/bioinformatics/bty560.

22. Stuart T, Butler A, Hoffman P, et al. Comprehensive Integration of Single-Cell Data. *Cell*. 2019;177(7):1888-1902 e21. doi:10.1016/j.cell.2019.05.031.

23. Stuart T, Butler A, Hoffman P, et al. Comprehensive Integration of Single-Cell Data. *Cell*. 2019;177(7):1888-1902.e21. doi:10.1016/j.cell.2019.05.031.

24. Aibar S, Gonzalez-Blas CB, Moerman T, et al. SCENIC: single-cell regulatory network inference and clustering. *Nat Methods*. 2017;14(11):1083-1086. doi:10.1038/nmeth.4463.

25. Efremova M, Vento-Tormo M, Teichmann SA, Vento-Tormo R. CellPhoneDB: inferring cell-cell communication from combined expression of multi-subunit ligand-receptor complexes. *Nat Protoc*. 2020;15(4):1484-1506. doi:10.1038/s41596-020-0292-x.

26. Travaglini KJ, Nabhan AN, Penland L, et al. A molecular cell atlas of the human lung from single-cell RNA sequencing. *Nature*. 2020;587(7835):619-625. doi:10.1038/s41586-020-2922-4.
